# Promoting scientific literacy in evolution through citizen science

**DOI:** 10.1098/rspb.2022.1077

**Published:** 2022-08-10

**Authors:** Miriam Brandt, Quentin Groom, Alexandra Magro, Dusan Misevic, Claire L. Narraway, Till Bruckermann, Anna Beniermann, Tom Børsen, Josefa González, Sofie Meeus, Helen E. Roy, Xana Sá-Pinto, Jorge Roberto Torres, Tania Jenkins

**Affiliations:** ^1^ Leibniz Institute for Zoo and Wildlife Research (IZW) im Forschungsverbund Berlin e.V., Alfred-Kowalke-Straße 17, Berlin, 10315, Germany; ^2^ Meise Botanic Garden, Nieuwelaan 38, 1860 Meise, Belgium; ^3^ Laboratoire Évolution and Diversité Biologique, UMR 5174, Université Toulouse III Paul Sabatier – Bâtiment 4R1, 118, route de Narbonne, 31062 Toulouse cedex 9, France; ^4^ ENSFEA-Univ. Toulouse, Castanet-Tolosan, France; ^5^ Université Paris Cité, Inserm, System Engineering and Evolution Dynamics, (CRI), 8bis Rue Charles V, 75004 Paris, France; ^6^ Learning Planet Institute, 8bis Rue Charles V, 75004 Paris, France; ^7^ Earthwatch Europe, Mayfield House, 256 Banbury Road, Oxford, OX2 7DE, UK; ^8^ Leibniz University Hannover, Schloßwender Str. 1, 30159 Hannover, Germany; ^9^ IPN – Leibniz Institute for Science and Mathematics Education, Olshausenstr. 62, 24118 Kiel, Germany; ^10^ Humboldt-Universität zu Berlin, Unter den Linden 6, 10099 Berlin, Germany; ^11^ Department of Planning, Aalborg University, A.C. Meyers Vænge 25, DK-2450 Copenhagen, SV, Denmark; ^12^ Institute of Evolutionary Biology (CSIC-Universitat Pompeu Fabra), Passeig Maritim Barceloneta 37-49, 08003 Barcelona, Spain; ^13^ UK Centre for Ecology and Hydrology, Benson Lane, Crowmarsh Gifford, Oxfordshire, OX10 8BB, UK; ^14^ Research Centre Didactics and Technology in the Education of Trainers, Campus Universitário de Santiago, University of Aveiro, Aveiro, Portugal; ^15^ La Ciència Al Teu Món, SciComm Association, Calle de Trafalgar 48, 08010 Barcelona, Spain; ^16^ University of Geneva, Science II, Quai Ernest Ansermet 30, 1205 Geneva, Switzerland

**Keywords:** evaluation, evolution misconceptions, education, learning, public participation in scientific research

## Abstract

Evolutionary understanding is central to biology. It is also an essential prerequisite to understanding and making informed decisions about societal issues such as climate change. Yet, evolution is generally poorly understood by civil society and many misconceptions exist. Citizen science, which has been increasing in popularity as a means to gather new data and promote scientific literacy, is one strategy through which people could learn about evolution. However, despite the potential for citizen science to promote evolution learning opportunities, very few projects implement them. In this paper, we make the case for incorporating evolution education into citizen science, define key learning goals, and suggest opportunities for designing and evaluating projects in order to promote scientific literacy in evolution.

## 1. Introduction

In a society fundamentally shaped by science and technology, scientific literacy is crucial in order to respond in a meaningful way to issues that pervade daily life and political actions. One scientific field for which this ‘everyday working knowledge of science’ [1] is particularly important is evolution. Evolutionary processes shape all aspects of the natural world [2], and many of the complex global challenges humanity is facing, such as human health (e.g. zoonotic diseases [3], antibiotic resistance [4], human microbiome [5]), food security [6] and biodiversity loss [7] are based on evolutionary processes. Furthermore, evolution has been applied in many fields outside biology, e.g. forensics [8], software development [9] and architecture [10]. Limited understanding of evolution can profoundly impair one's ability to make rational decisions on societal issues [11]. For instance, the COVID-19 pandemic has demonstrated that evolution impacts our daily lives: a genetic sequence inherited from Neanderthals increases the odds of hospitalization [12] and SARS-CoV-2's evolutionary history and ongoing evolution informs vaccine development [13].

Despite its importance, evolution is generally poorly understood [14] and is not always accepted by the public [15]. Understanding evolution requires more than just the learning of ‘facts’—promotion of scientific literacy in evolution is necessary. Scientific literacy involves being able to explain phenomena scientifically, evaluate and design scientific inquiry, interpret data and evidence scientifically [16]. These require knowledge about the content of science (content knowledge), an understanding of scientific methods (procedural knowledge) and insights into how scientific knowledge is created (epistemic knowledge) [17]. In addition, the ability to use scientific knowledge and reasoning in different situations (knowledge application) is required [18].

Citizen science (CS), defined here as the participation of non-professional scientists in research, is a suitable tool for increasing scientific literacy [19]. Indeed, CS projects provide an excellent context for learning: often rooted in real-life contexts, presenting cognitive challenges, and offering participation in hands-on scientific tasks [20]. These aspects are generally acknowledged as being essential ingredients of active learning [21], suggesting CS can achieve educational impacts. Unfortunately, its learning dimension is underexplored [22], and evidence for learning outcomes is scant [19,23,24].

Despite the centrality of evolution to biology, very few biology CS projects frame their activities in an evolutionary context. For example, of the 1603 projects on the CS platform SciStarter (https://scistarter.org/, as of June 2022), 672 are in ‘ecology and environment’, while only 14 mention evolution. We consider this to be a missed opportunity for promoting scientific literacy in evolution.

Here, we define different types of learning outcomes, describe challenges in promoting scientific literacy in evolution through CS, give recommendations, provide guidelines on how to design for learning, and evaluate the outcomes. While we focus on CS in evolution, many of our recommendations on creating and evaluating learning opportunities are more generally applicable to other fields of biology and CS more broadly. For example, learning opportunities for epistemic knowledge can be included in any CS project, regardless of the investigated topic (see section ‘Creating learning opportunities in citizen science projects’). In addition, evaluation of learning outcomes in CS projects is currently rare. The suite of instruments that we suggest (see section ‘Evaluating learning outcomes in citizen science projects') could be applied to any CS project looking to assess participant learning. To effectively apply our recommendations to other fields, researchers need to be clear about the learning outcomes they want to achieve and consider why participants might be interested in their project.

## 2. Key learning goals for scientific literacy in evolution

Including an educational dimension in a CS project requires being clear about its scientific goals and possible learning outcomes. Right from the beginning, aligning these outcomes with project goals and educational opportunities in the design is essential [24]. To increase scientific literacy, four key learning goals are crucial: content knowledge, procedural knowledge, epistemic knowledge and knowledge application (table 1). For the many other worthwhile outcomes of CS projects, such as relating to behaviour, interest, self-efficacy and motivation, we refer the reader to other frameworks [24,25]. Next, we explore the importance of the four learning goals in the context of evolution.

**Table 1.** Examples covered by the four learning goals.

learning goal	examples
content knowledge	phenotypic variation; heritability of traits; selective pressure; adaptation
procedural knowledge	observing variability within a population; recording changes in a certain trait over time; aligning DNA sequences; formulating hypotheses and designing studies
epistemic knowledge	meaning of considering evolution as a ‘theory’; understanding that scientific knowledge is constantly changing through the addition of new evidence; understanding that science is embedded in society and influenced by cultural norms
knowledge application	understand, be able to discuss and/or make informed decisions about issues such as: the emergence of new SARS-CoV-2 strains and the impact of COVID-19 vaccines; the importance of crop biodiversity for food security; the impact of invasive species

### (a) Content knowledge

Developing a good understanding of evolution and using evolutionary knowledge to explain biological scenarios requires a grasp of key concepts. Evolutionary theory rests on a network of foundational disciplines ranging from genetics to ecology and geology. Thus, understanding evolution requires synthesis and coordination of multiple perspectives, which is a challenge for learning and teaching [26]. This starts with understanding key concepts, such as ‘adaptation’, ‘variation’ and ‘selective pressure’, and words like ‘theory’ or ‘fitness’ (see ‘Communication issues'), in order to structure the acquired knowledge [27].

### (b) Procedural knowledge

Within CS projects, participants may be more familiar with certain types of procedural knowledge such as species identification, whereas they may be less familiar with others, such as analysing data and discussing evidence [28]. Procedural knowledge is important in the context of evolution because many evolutionary processes cannot be directly observed or subjected to experimentation, either because they took place in the past, and/or because they occur over large temporal and spatial scales, which may hinder understanding [29].

### (c) Epistemic knowledge

CS projects may also constitute a way to increase public understanding of the nature of science, that is, the characteristics of scientific knowledge and the way it is produced [30]. Research results are initially uncertain, can be contradictory, and are not definitive. In order to interpret research results appropriately a differentiated view of findings—from new, still uncertain findings, to accepted facts—is essential. This is especially pertinent with regard to evolution, as scientific debate over new results on evolutionary mechanisms is sometimes interpreted as disagreement within the scientific community on whether or not evolution happens [31]. Indeed, it has been shown that understanding the nature of science increases students' acceptance of evolution [32].

### (d) Knowledge application

Scientific literacy in evolution is required for citizens to understand how the world works as a system and inform decisions regarding global challenges [3,33]. It is therefore important that they are able to apply evolutionary knowledge learned in projects to other situations [34].

Although CS projects may promote learning across all four dimensions of scientific literacy, it is unlikely to be possible to address them all equally well. Which learning goals can realistically be achieved depends on the specific topic, methodology and project set-up. We will elaborate on how to create learning opportunities on evolution, after considering some important barriers to learning about evolution.

## 3. Barriers to learning evolution

Identifying barriers to learning evolution is essential in order to design for learning. Here, we describe three types of barriers: misconceptions, conflicts with established culture and values, and communication issues.

### (a) Misconceptions about evolution

A key challenge for scientists trying to increase scientific literacy in evolution is that important details of evolution by natural selection are often misinterpreted. For example, many people are not aware that mutations are random and have a range of effects; that the potential for adaptability is not unbounded; nor that ‘survival of the fittest’ refers to how organisms compare to each other, rather than some absolute fitness metric. Indeed, misconceptions are frequent and widespread across different demographic groups, including young students, teachers and the general public [35–37].

In evolution, concepts that are abstract or counterintuitive include the difficulty to conceive of the spatial and temporal scales over which evolution occurs, probability and randomness [38,39]. In addition, understanding evolution requires linking a number of complex concepts and misconceptions about any one of them will impact the understanding of the others [40].

Misconceptions exist even among those that accept evolution [41] and are remarkably resilient to instruction [42]. Additionally, they can be context dependent: students may provide correct explanations for trait gain in one organism but fail to transfer that explanation to another species [43].

### (b) Conflicting culture and values

Educational approaches that focus on increasing knowledge about evolution might fail if they conflict with the culture and values of participants [16]. As public attitudes toward evolution are sometimes negative [44] they should be considered a key factor when implementing projects on evolution. Probably the most persistent example for a conflict is that between religion and acceptance of evolution [45]. This conflict predominantly occurs between evolution and some denominations of Christianity and Islam [38] and its extent is highly country dependent [46].

Acceptance of evolution is also influenced by the total number of years spent in education [38], understanding of nature of science [47], attitudes towards science [48], knowledge/understanding of evolution [49], and gross domestic product per capita [50]. Additionally, there is still a debate about the relationship between acceptance and actual understanding of evolution with conflicting evidence for strong positive correlation [51], weak positive relationship [36,37,48], or no correlation at all [52].

### (c) Communication issues

Effective communication in CS projects is challenging as scientists are predominately trained to communicate using specialized terminology. Moreover, some evolutionary terminology has different meanings in the scientific community and in colloquial language [53]. For example, ‘evolution’ is used colloquially to mean ‘change over time’, stripping it of its scientific meaning [54]. Similarly, colloquially, ‘theory’ is something unproven [55], and ‘selection’ implies a conscious selector [56]. Finally, translation between different languages may introduce an additional layer of ambiguity. In Roman languages there is no word for ‘fit’, and in Serbian and French, fitness is often translated as ‘adaptive value’ which could unintentionally imply an adaptationist view.

## 4. Creating learning opportunities in CS projects with a focus on evolution

Despite the huge potential for CS to achieve learning goals [19], this dimension is often underexploited [22]. One indirect way of achieving learning is to raise the level of participation that the project offers [57]. However, offering higher levels of engagement, such as the additional opportunity to analyse data, does not necessarily increase the learning outcomes [58]. Therefore, to achieve broader educational impacts, increasing the level of engagement will not suffice. When CS project initiators decide to include a learning dimension, their efforts will yield better results if learning goals and opportunities are clearly defined from the outset. We now consider how existing projects have designed learning opportunities in evolution, focusing on the learning dimensions defined above. Table 2 provides suggestions on how to promote learning opportunities of evolution in CS projects, that researchers can choose from, tailored to the goals and circumstances of the project (e.g. resources and expertise of the team).

**Table 2.** Examples of opportunities to promote learning on evolution in CS projects. The selection of measures implemented will depend on the goals and circumstances of the project.

opportunity	implementation examples	considerations to improve learning when implementing in context of a project
curriculum-based activities	implement activities with school classes	consider collaborating with teachers and education researchers [59]
		align educational activities with national curricula to make them attractive for educators [60]
		identify the requirements and expectations of teachers and students [60], perhaps with the help of a logic model [61]
co-design of the project	involve participants in developing research questions, study design, data analysis and/or communication	consider co-design to broaden learning opportunities for epistemic knowledge and knowledge application [62–64]
		implement learning activities prior to or during co-creation processes [20], so participants can contribute meaningfully
		allow and value contributions for multiple experiences and backgrounds to enhance learning and ownership [65]
		engage participants in the design of outreach strategies [66] to promote positive attitudes
data collection, data analysis, understanding the nature of science	provide training resources to underpin data collection, data analysis and background context	explicitly teach participants about the steps of scientific inquiry [67]
		combine teaching the necessary skills with (i) evolutionary background to provide conceptual context [20] and (ii) explaining the value of rigorous data collection and analysis [68]
		encourage participant feedback to improve and develop the study methods [69]
		give participants the opportunity to engage in different tasks [70]
gamification	implement gamification of evolutionary content and/or of participation (i.e. achievement badges)	use gamification to sustain participant interest and to motivate people not intrinsically motivated to participate in learning opportunities [71,72]
		use gamification of participation to help participants develop a feeling of self-efficacy [73]
		be careful not to oversimplify information about evolution in games, as this may generate misconceptions [74]
communicating with participants	use uni-directional communication (e.g. emails, social media, website, field guides) as well as dialogue/social interactions (e.g. online, or in person at formal or informal meetings)	engage in active public relations work [75]
		acknowledge participants' contributions, as this helps to maintain their interest [64,76]
		show respect for differing cultural, religious and educational backgrounds of participants [32]
		share data, results, and information on how the data are used to evaluate potential evolutionary explanations [77,78]
		invest in creating social interactions, as these promote learning and positive attitudes towards science [79]
		refer participants to other projects in evolution to keep them engaged and increase learning outcomes [80]
		make content more accessible by explaining real-world relevance [20] and through storytelling [81]
		use clear language: be careful when using terms that have different meanings colloquially [55]
promoting peer-to-peer participant communication	use narrative story-telling by participants (e.g. photo diaries), online communication (e.g. social media, blogs), formal and informal meetings	have participants communicate knowledge from long-term memory as this active application increases learning [82]
		reflect with participants on their peer-to-peer communication to avoid spread of misconceptions
		discuss with participants which points they communicate, including relevant background [83]
		encourage more advanced participants to teach beginners (near-peer teaching) to benefit learning for both [84]
		support critical thinking by encouraging participants to discuss how their findings build evolutionary knowledge [77]

### (a) Creating learning opportunities for content and procedural knowledge

Simply presenting concepts or theories, and describing the scientific methods applied to evolutionary research, cannot be assumed to automatically increase citizen scientists' understanding of evolution [85]. To foster content and procedural knowledge, projects should provide active learning situations, supported by educational resources adapted to misconceptions, cultures and values of different groups. This occurred in ‘Evolution MegaLab’ [86] which mobilized 6461 registered participants to survey colour morphs of banded snails to map climate change effects. Communication resources explaining the evolutionary background of morph variation were adapted to different target audiences, and participants had immediate feedback on their results. As a result, it helped participants grasp the notion that evolution can be observed directly.

Likewise, in the ‘1000 Gardens’ project [87] 2492 registered participants engaged in an artificial selection experiment that provided data on the performance of soya bean genotypes at different latitudes. The theoretical background was explained in the context of the broader experimental design, and participants performed a small part of the experiment in their garden. At the end of the project, the results and conclusions of the project were shared with participants [88].

Such hands-on involvement also contributes to the acquisition of skills and methods relevant to studying evolution (procedural knowledge). For instance, in ‘*Melanogaster:* catch the Fly!’ [89], participants (about 320 school students to date) have the opportunity to learn about bioinformatics and use these tools to analyse evolution at the genomic level.

### (b) Creating learning opportunities for epistemic knowledge

While participants may gain increased content and procedural knowledge, there is no consensus in the literature on if this leads to an increased understanding of the nature of science [90], or influences people's acceptance of evolution [32]. Participants grasp major aspects of the nature of science more easily when they conduct experiments [91]. However, this may not be enough [92], and resources specifically designed to address distinct components of the nature of science are needed. The ‘*Pieris* project’ with participants from 30 US states and 32 different countries [93], examines how organisms respond to environmental change, provides information about the diversity of methods employed to infer the history of cabbage white butterfly populations, and the empirical evidence supporting their inferences on the history of invasion. Furthermore, it addresses the question of how to deal with uncertainty, illustrating that science is open to revision in the light of new evidence.

### (c) Creating learning opportunities to foster knowledge application

To achieve a larger impact on scientific literacy, projects with a focus on evolution should empower participants to apply acquired knowledge to new situations by highlighting its broader relevance and encouraging further engagement with other projects or communities. Many projects include blogs, or are connected to social platforms, fostering interaction with a broad spectrum of perspectives beyond the project's central subject [94]. ‘SquirrelMapper’ [95], a project that examines rapid adaptation to a changing environment in eastern grey squirrels, and which has amassed approximately 25 000 participants, goes even further. It gives citizen scientists the opportunity to apply their acquired knowledge to another CS project regarding the management of grey squirrels in cities, promoting engagement with other sectors of society.

### (d) Designing learning opportunities to address misconceptions

The first step for dealing with misconceptions is to anticipate them [96]. The KAEVO 2.0 instrument [36] can be used by CS projects to assess knowledge and misconceptions about evolution [97]. After which, rather than simply communicating facts, projects need to encourage participants to exert critical thinking [32]. Thus, project initiators should give participants the opportunity to test their prior knowledge by offering situations that challenge likely misconceptions [96]. As misconceptions are tenacious, it is important to revisit them frequently and to assess the validity of the participants' understanding (including by self-assessment). Social interactions that give space for conflicting viewpoints and communication, in addition to being beneficial for learning, also help to overcome misconceptions [98]. As such, it is useful for initiators to implement an array of approaches to improve interaction and offer choices that accommodate participants’ differences. This could also increase engagement and fidelity that reinforce learning [99].

## 5. Evaluating learning outcomes in evolution in citizen science projects

It is not sufficient to only design to promote scientific literacy as this does not guarantee uptake by participants. For instance, if learning opportunities are not at the right level they may not be used, since both over-straining and demanding too little is discouraging [100]. To find out if approaches are effective, we need to assess the learning outcomes achieved.

Although there are opportunities for learning in CS, the evidence of learning outcomes, especially with respect to scientific literacy, is sparse [23,24]. For example, in a non-exhaustive literature search of SciStarter, Google Scholar and Web of Science, we identified 58 CS projects on evolution, 38 of which (65%) claimed to have a learning outcome. Of those, only 10 (26%) actually evaluated it. Out of the five projects described above as providing learning opportunities (Evolution MegaLab, 1000 Gardens, *Melanogaster*: catch the fly, Pieris and SquirrelMapper), two evaluate for learning outcomes (JR Torres, J Gibbs 2022, personal communication).

Most CS projects aiming to promote participants' scientific literacy tend to only measure content knowledge [101]. However, a number of methods and instruments to evaluate the other learning outcomes exist (table 3), as well as a shared framework to measure individual learning outcomes from participation [24]. The selection of tools used will depend on the resources available for evaluation and the skillset of the project team, which could be augmented by interdisciplinary collaboration (e.g. with education scientists).

**Table 3.** Examples of measurement instruments and approaches to evaluate dimensions of scientific literacy. The selection of measurement instruments used will depend on the goals and circumstances of the project.

name of measurement instrument or method	evaluated construct
**content knowledge**^a^	
Assessing COntextual Reasoning about Natural Selection (ACORNS) [102]	understanding of natural selection, adaptive change
Concept Inventory of Natural Selection (CINS) [103]	natural selection
Knowledge About EVOlution 2.0 (KAEVO 2.0) [36,97]	several micro- and macro- evolutionary concepts
**procedural knowledge**	
assessing experimental design [104]	planning a scientific study and sampling design
FOrmal Reasoning Test (FORT) [105]	scientific reasoning abilities
Scientific Reasoning Scale (SRS) [106]	abilities for evaluating scientific findings
participant observation [107]	group processes in knowledge production
**epistemic knowledge**	
Connotative Aspects of Epistemological Beliefs (CAEB) [108]	epistemological beliefs
views of Nature of Scientific Inquiry (NOSI views) [109]	understanding nature of scientific inquiry
Student Understanding of Science and Scientific Inquiry (SUSSI) [110]	understanding science and scientific inquiry
Views About Scientific Inquiry (VASI) [111]	understanding scientific inquiry
Views of Nature of Science (VNOS) [30]	understanding nature of science
**knowledge application**	
Quantitative Assessment of Socio-Scientific Reasoning (QuASSR) [112]	socioscientific reasoning
participant observation [107]	application of acquired knowledge in discussions

^a^For a full review of instruments that measure evolution understanding see [38,113].

### (a) Recommendations for choosing and designing evaluation instruments

When selecting evaluation instruments three key aspects need to be considered:

#### (i) Depth and type of evaluation

Evaluations can be quantitative, issued as closed questionnaires (e.g. self-reporting or tests [114]); or qualitative, performed as open questionnaires or semi-structured interviews [115], participant observation [107], focus groups, photo diaries and the study of narratives [116].

#### (ii) Applicability to the study population

In quantitative evaluation, instruments are designed, applied and validated for particular study populations and therefore may not be directly transferable. If no prior validation exists for the study population, a small pilot is recommended before the start of the project [115].

#### (iii) Communicating evaluation goals and process

It is necessary to explain to participants the importance of evaluation and its requirements. Keep the measures as short as possible, and focus on the dimensions of scientific literacy your project targets. Goals must be made clear from the start and codes of ethics followed [117]. Co-evaluation, where project participants are involved in designing the project evaluation strategy, can be a useful tool to overcome participation barriers [101].

## 6. Balancing scientific goals with designing for learning and evaluation: challenges and benefits

Including a learning dimension in a CS project might be seen as a trade-off to the primary interests of the project initiator to achieve scientific outcomes and academic excellence [118]. Furthermore, project initiators often lack knowledge, incentive and resources to design for learning [19]. Yet, including learning opportunities can provide tangible benefits. Learning is an important factor for continuing motivation of participants [119], which in turn strongly affects data quality and quantity, as well as the project's societal impact through participants' willingness to advocate the topic [120,121].

Achieving learning outcomes can lead to societal impacts, which are increasingly recognized as central in research policy [122] and an important goal of academic researchers [123]. Many policymakers and funding agencies are already requiring CS projects to design and assess their learning outcomes [57], and this request is likely to be met by increasing financial support. For example, the SquirrelMapper project initiators were equally interested in the educational and biological dimensions of the project and developed the educational aspect for 10 years without funding. The project now has major funding for both dimensions, which are advanced simultaneously by an interdisciplinary team (J Gibbs 2022, personal communication). As such, clear benefits exist of designing for and evaluating learning outcomes.

Interdisciplinary collaborations can also contribute to solving the dilemma of having to divert resources to aspects that project initiators may not see as focal. Hence, collaboration between evolutionary biologists and education scientists/educators is suggested from the beginning of the project [124], resulting in a win–win situation. Indeed, for education researchers it may be scientifically rewarding to apply their expertise to this new learning context. However, interdisciplinary work requires open-mindedness, empathy, trust, transparency of different objectives, and an effort to develop mutual understanding [125] to create synergies between the different perspectives, values and norms involved.

## 7. Conclusion

In this paper, we argue that there is great potential for CS as a tool for evolution education. However, CS is not fully exploited as a research or educational tool by evolutionary biologists. Many projects either have no explicit learning goals, or if they do, it is often assumed that learning will happen by default when people participate in project activities. In reality, a positive effect on scientific literacy in evolution can only be achieved if projects are purposely designed and evaluated for learning outcomes. For this, we would like to encourage evolutionary biologists to develop CS projects in evolution, and actively engage with education scientists/educators who can contribute expertise on increasing scientific literacy in evolution.

## Data Availability

This article has no additional data.
